# Supplementary material for: An Autoimmunity-associated allele of PTPN22 enhances the innate immune response to promote protection against acute coronavirus infection
Source: bioRxiv. 2025 Jul 18:2025.07.15.664967. Preprint. [Version 1] doi: 10.1101/2025.07.15.664967 (PMC12338618; doi:10.1101/2025.07.15.664967)
Supplement: Supplementary file 1 [file NIHPP2025.07.15.664967v1-supplement-1.pdf]

bioRxiv preprint doi: https://doi.org/10.1101/2025.07.15.664967; this version posted July 18, 2025. The copyright holder for this preprint (which was not certified by peer review) is the author/funder, who has granted bioRxiv a license to display the preprint in perpetuity. It is made available under aCC-BY 4.0 International license.

Supplementary Materials

Fig. S1

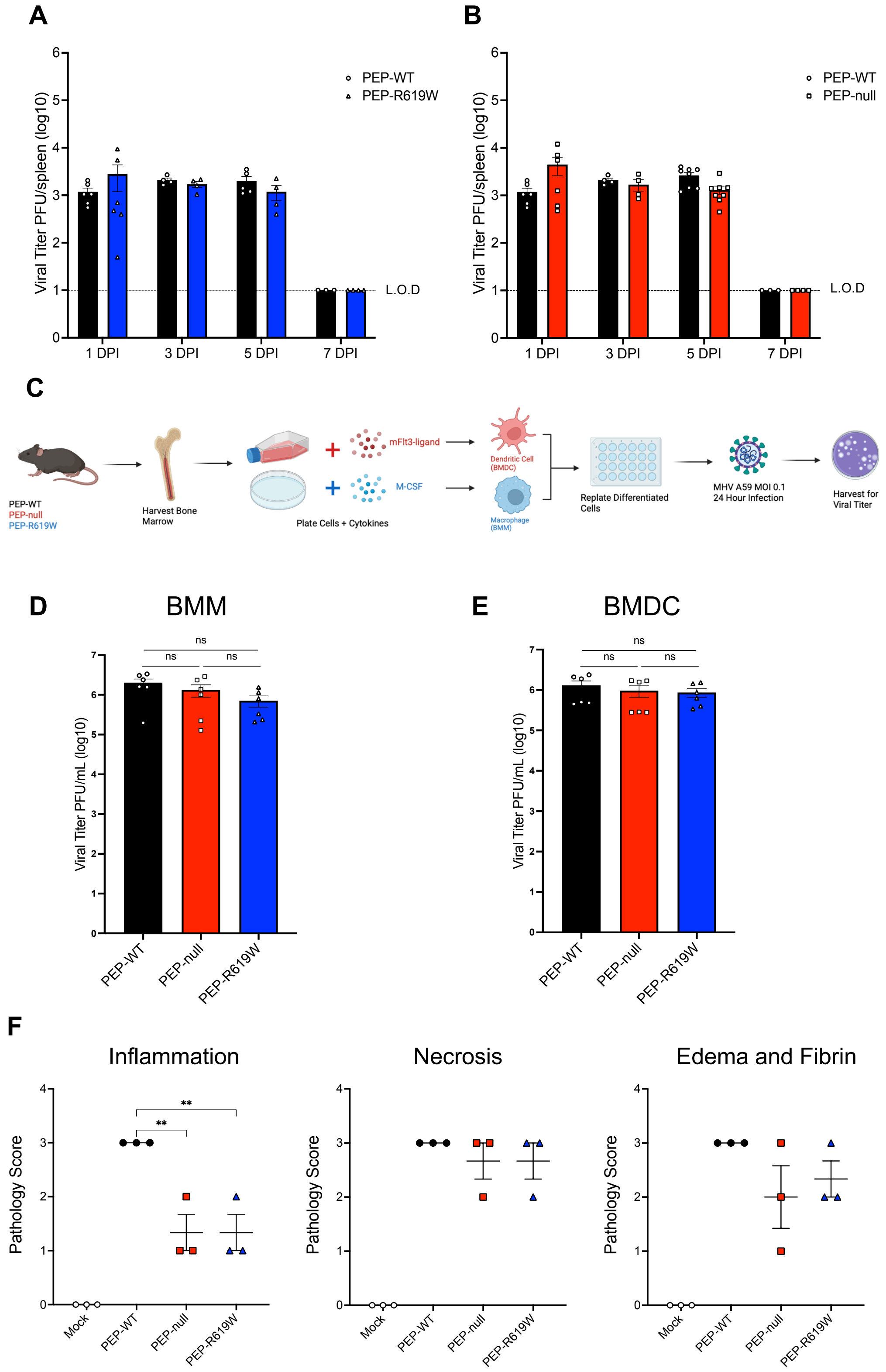

bioRxiv preprint doi: https://doi.org/10.1101/2025.07.15.664967; this version posted July 18, 2025. The copyright holder for this preprint (which was not certified by peer review) is the author/funder, who has granted bioRxiv a license to display the preprint in perpetuity. It is made available under aCC-BY 4.0 International license.

Figure S1: PEP-R619W does not impact splenic titer or restrict viral replication in myeloid cells.

To measure viral burden, spleen homogenates from infected (5e2 PFU MHV A59) PEP-WT, PEP-null, and PEP-R619W mice were used for plaque assay (A, B). Experimental schematic for deriving bone marrow-derived macrophages (BMMs) and bone marrow-derived dendritic cells (BMDCs) (C). The viral titer was quantified via Plaque Assay for BMMs (D) and BMDCs (E). Liver histopathology scores of individual parameters (inflammation, necrosis, and edema/fibrin) at 3 dpi following 1e5 PFU MHV A59 infection are shown (F). For whole spleen titer (A, B), each symbol represents an individual mouse. The dashed line indicates the limit of detection (L.O.D.). Certain PEP-WT mice are represented in both graphs, as some experiments had all three genotypes, whereas others contained only PEP-WT vs. PEP-null or PEP-R619W. Combined data from 2 independent experiments, multiple unpaired T-Tests, PEP-WT vs PEP-R619W, PEP-WT vs PEP-null, at each time point. For BMM and BMDC titer, each MHV A59 infection was performed at MOI = 0.1. Data are shown from two independent experiments with 3 technical replicates per experiment. For histopathological analysis, each dot represents an individual mouse, with p-values from One-way ANOVA with Tukey's Multiple Comparisons shown, *$p<0.05$, **$p<0.01$.

bioRxiv preprint doi: https://doi.org/10.1101/2025.07.15.664967; this version posted July 18, 2025. The copyright holder for this preprint (which was not certified by peer review) is the author/funder, who has granted bioRxiv a license to display the preprint in perpetuity. It is made available under aCC-BY 4.0 International license.

Fig. S2

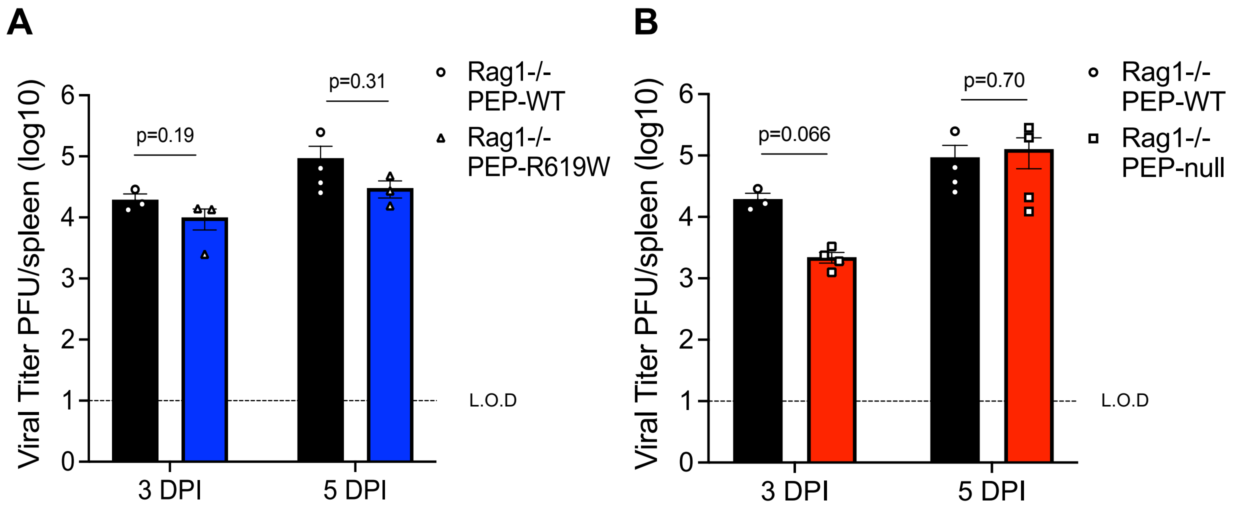

Figure S2: Splenic titers are unchanged in Rag1-/- mice regardless of PEP genotype.

To measure viral burden, liver homogenates from infected (5e2 PFU) Rag1-/- PEP-WT (black), Rag1-/- PEP-null (red), and Rag1-/- PEP-R619W (blue) mice were used for plaque assay (A, B). Each symbol represents an individual mouse. The dashed line indicates the limit of detection (L.O.D.). Certain Rag1-/- PEP-WT mice are represented in both graphs, as some experiments had all three genotypes, whereas others contained only Rag1-/- PEP-WT vs Rag1-/- PEP-null or Rag1-/- PEP-R619W. Multiple unpaired T-Tests, PEP-WT vs PEP-R619W, PEP-WT vs PEP-null, at each time point.

bioRxiv preprint doi: https://doi.org/10.1101/2025.07.15.664967; this version posted July 18, 2025. The copyright holder for this preprint (which was not certified by peer review) is the author/funder, who has granted bioRxiv a license to display the preprint in perpetuity. It is made available under aCC-BY 4.0 International license.

34 **Fig. S3**

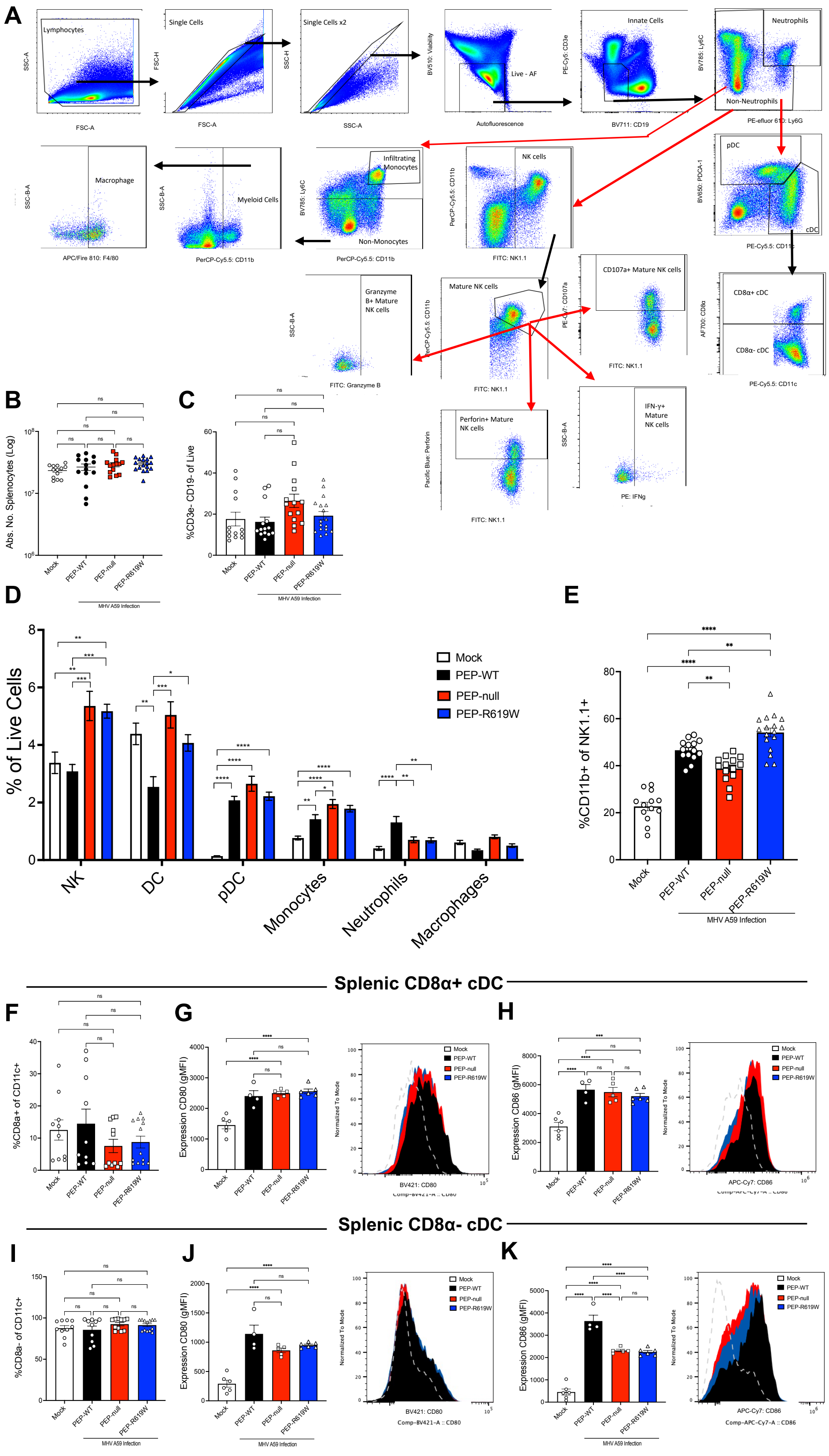

bioRxiv preprint doi: https://doi.org/10.1101/2025.07.15.664967; this version posted July 18, 2025. The copyright holder for this preprint (which was not certified by peer review) is the author/funder, who has granted bioRxiv a license to display the preprint in perpetuity. It is made available under aCC-BY 4.0 International license.

Figure S3: PEP-R619W increases the splenic innate immune population.

Representative gating strategies to identify splenic immune cell populations of interest (A). Populations are labeled on flow cytometry plots, where black arrows indicate the down-gating of the population from which they are drawn from, and red arrows indicate the down-gating from one population to multiple populations. Following PBS injection (Mock, white bar, circle) or 5e4 PFU MHV A59 infection (i.p) in PEP-WT (black bar, circle), PEP-null (red bar, square), PEP-R619W (blue bar, triangle) mice, at 3 dpi the spleen was harvested and antibody stained for flow cytometric analysis. The absolute count of splenocytes for all genotypes (B). Frequencies (%) of CD3e- CD19- of Live cells (C). Frequency (%) of several innate cells of Live cells is shown (D). The gating strategy for all innate subsets is: Lymphocytes> Single cell x2> autofluorescent-> Live> CD3e- CD19-> Ly6G- Ly6C+/- (non-neutrophils). NK Cells: innate subsets> NK1.1+. DCs: innate subsets> CD11c+ PDCA-1-. pDCs: innate subsets> PDCA-1+ CD11c+/-. Monocytes: innate subsets> Ly6Chi CD11b+. Macrophage: innate subsets> Ly6C- CD11b+/-> CD11b+> F4/80+. Neutrophils: Lymphocytes> Single cell x2> autofluorescent-> Live> CD3e- CD19-> Ly6G+ Ly6C+. Frequency (%) of CD11b+ (mature) NK cells (E). Frequency (%) Splenic CD8α+ cDCs (F) and expression (gMFI) of CD80 (G) and CD86 (H) with representative histograms to the right. Frequency (%) CD8α- cDCs (I) and expression (gMFI) of CD80 (J) and

bioRxiv preprint doi: https://doi.org/10.1101/2025.07.15.664967; this version posted July 18, 2025. The copyright holder for this preprint (which was not certified by peer review) is the author/funder, who has granted bioRxiv a license to display the preprint in perpetuity. It is made available under aCC-BY 4.0 International license.

CD86 (K) with representative histograms to the right. Quantification of flow cytometric data was pooled from 3 independent experiments. Each symbol represents an individual mouse. Mock mice are a mixture of all 3 genotypes. p-values from One-way ANOVA with Tukey's Multiple Comparisons are shown for each panel. *p<0.05, **p<0.01, ***p<0.001, ****p<0.0001.

bioRxiv preprint doi: https://doi.org/10.1101/2025.07.15.664967; this version posted July 18, 2025. The copyright holder for this preprint (which was not certified by peer review) is the author/funder, who has granted bioRxiv a license to display the preprint in perpetuity. It is made available under aCC-BY 4.0 International license.

60 **Fig. S4**

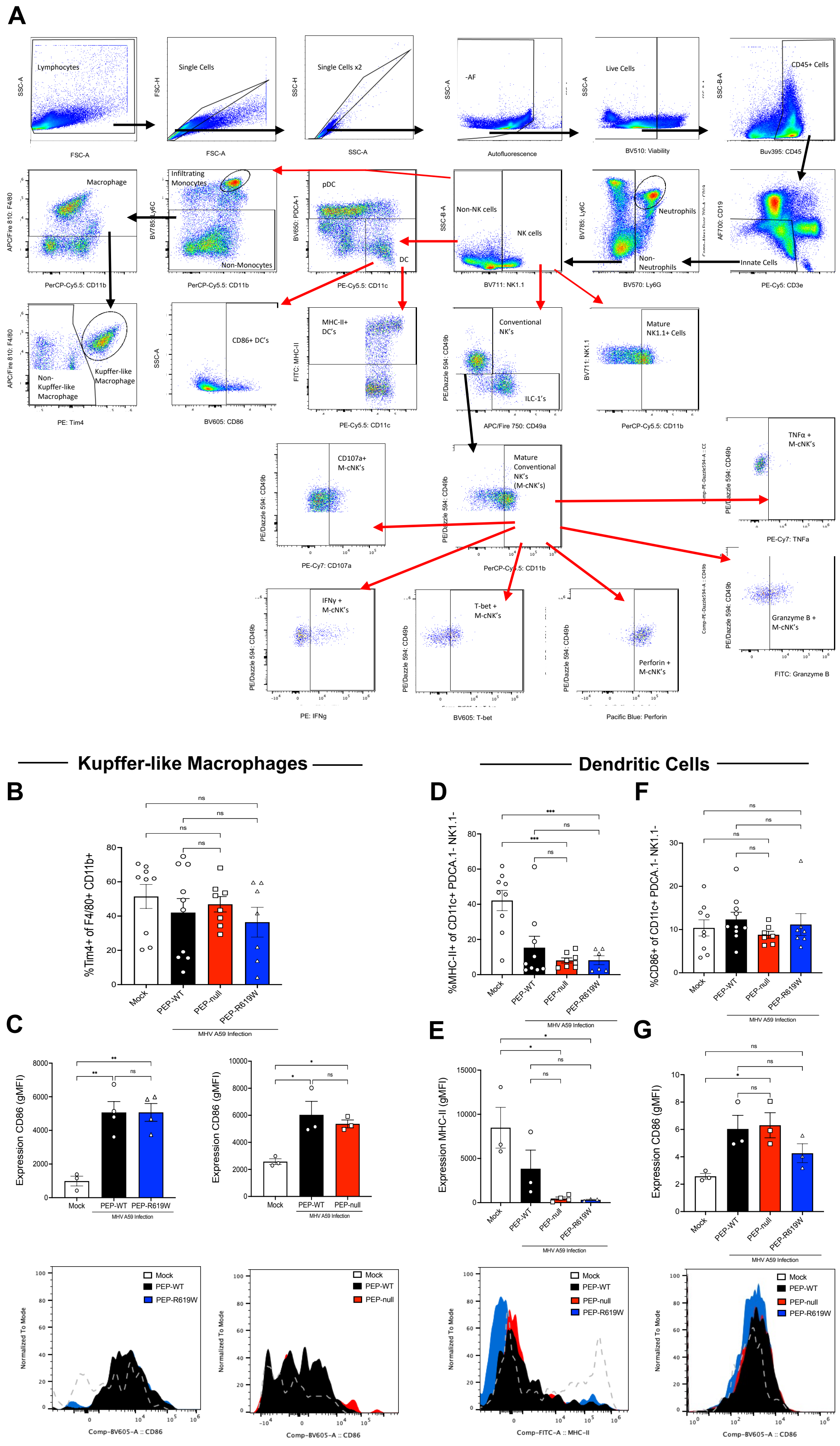

bioRxiv preprint doi: https://doi.org/10.1101/2025.07.15.664967; this version posted July 18, 2025. The copyright holder for this preprint (which was not certified by peer review) is the author/funder, who has granted bioRxiv a license to display the preprint in perpetuity. It is made available under aCC-BY 4.0 International license.

Figure S4: Representative gating strategy for different liver immune cell populations and additional quantification of immune cells.

Representative gating strategies to identify liver immune cell populations of interest presented in Figure 3 (A). Populations are labeled on flow cytometry plots, where black arrows indicate the down-gating of the population from which they are drawn from, and red arrows indicate the down-gating from one population to multiple populations. Following PBS injection (Mock, white bar, circle) or 1e5 PFU MHV A59 infection (i.p) in PEP-WT (black bar, circle), PEP-null (red bar, square), PEP-R619W (blue bar, triangle) mice, at 3 dpi the liver was perfused, harvested, and antibody stained for flow cytometric analysis. Tim4+ Macrophages (Kupffer-like cells) frequency (%) (B) and expression of CD86 (gMFI) in Tim4+ Macrophages (C), with representative histograms below. The first plot (left) comparing Mock, PEP-WT, and PEP-R619W and the second plot (right) comparing Mock, PEP-WT, and PEP-null are representative of two separate independent experiments. The gating strategy for Kupffer-like (Tim4+ macrophages) is: Lymphocytes> Single cell x2> autofluorescent-> Live> CD45+> CD3e- CD19-> Ly6G- Ly6C+/- (non-neutrophils)> NK1.1-> Ly6C- CD11b+/-> F4/80+ CD11b+> Tim4+. MHC-II+ DCs frequency (%) (D) and expression of MHC-II (gMFI) in DCs (E) with a representative histogram below. CD86+ DCs frequency (%) (F) and expression of CD86 (gMFI) in DCs (G) with a

bioRxiv preprint doi: https://doi.org/10.1101/2025.07.15.664967; this version posted July 18, 2025. The copyright holder for this preprint (which was not certified by peer review) is the author/funder, who has granted bioRxiv a license to display the preprint in perpetuity. It is made available under aCC-BY 4.0 International license.

representative histogram below. The gating strategy for MHC-II+ DCs or CD86+ DCs is: Lymphocytes> Single cell x2> autofluorescent-> Live> CD45+> CD3e- CD19-> Ly6G- Ly6C+/- (non-neutrophils)> NK1.1-> CD11c+ PDCA-1-> MHC-II+ or CD86+. Quantification of flow cytometric data was pooled from 2 independent experiments. Each symbol represents an individual mouse. Mock mice are a mixture of all 3 genotypes. p-values from One-way ANOVA with Tukey's Multiple Comparisons are shown for each panel. *p<0.05, **p<0.01, ***p<0.001, ****p<0.0001.

bioRxiv preprint doi: https://doi.org/10.1101/2025.07.15.664967; this version posted July 18, 2025. The copyright holder for this preprint (which was not certified by peer review) is the author/funder, who has granted bioRxiv a license to display the preprint in perpetuity. It is made available under aCC-BY 4.0 International license.

Fig. S5

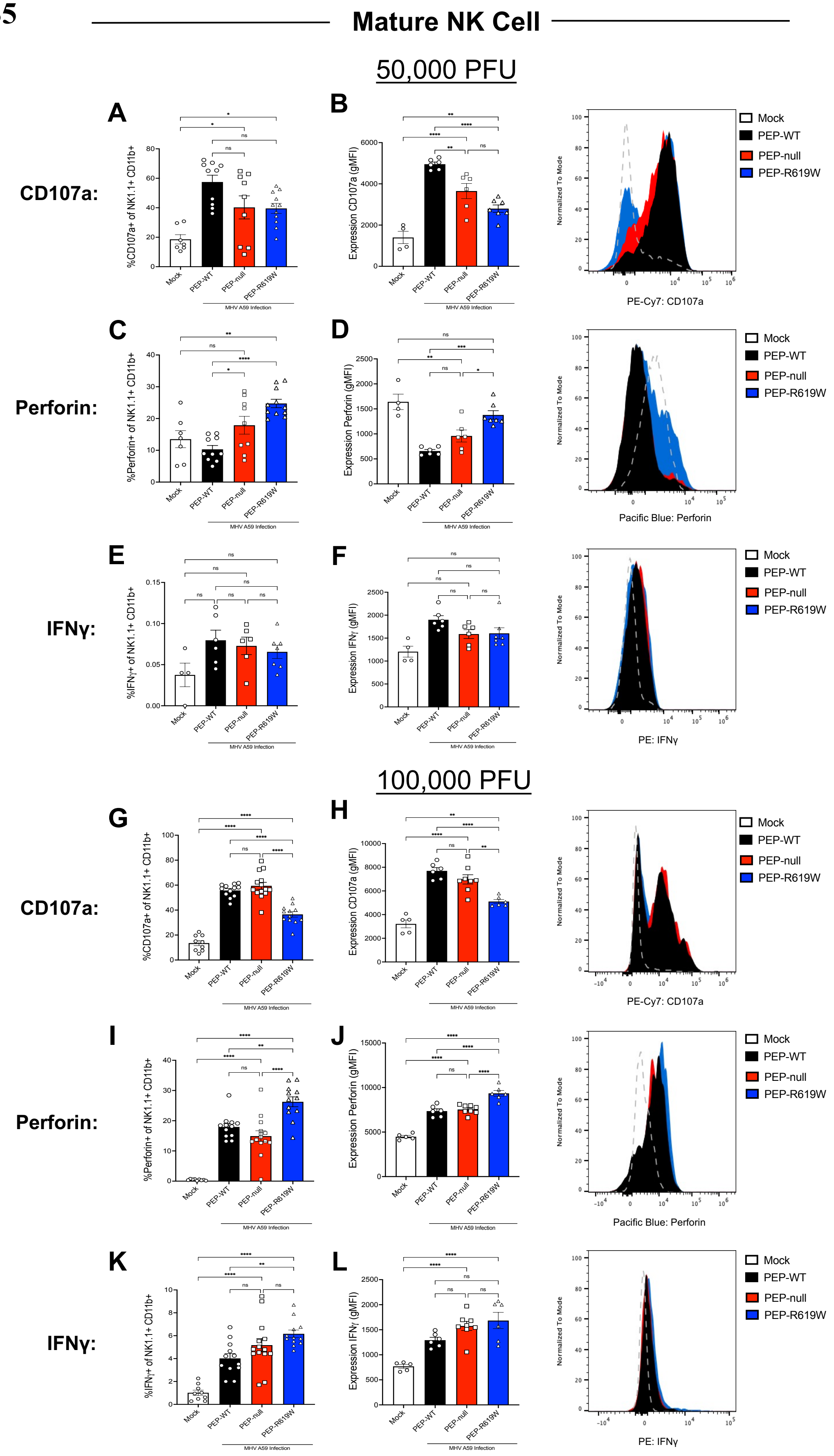

bioRxiv preprint doi: https://doi.org/10.1101/2025.07.15.664967; this version posted July 18, 2025. The copyright holder for this preprint (which was not certified by peer review) is the author/funder, who has granted bioRxiv a license to display the preprint in perpetuity. It is made available under aCC-BY 4.0 International license.

Figure S5: Infection dose-dependent impact on splenic mature NK function.

Following PBS injection (Mock, white bar, circle) or 5e4 PFU (A-F) or 1e5 PFU (G-L) MHV A59 infection (i.p) in PEP-WT (black bar, circle), PEP-null (red bar, square), and PEP-R619W (blue bar, triangle) mice, at 3 dpi the spleen was harvested, surface and intracellularly antibody stained for flow cytometric analysis. Frequency (%), expression (gMFI), and representative histogram to the right of CD107a (A-B, G-H), Perforin (C-D, I-J), and IFNγ (E-F, K-L) in Mature NK cells, for 5e4 PFU and 1e5 PFU MHV A59 infections, respectively. The gating strategy for Mature NK cells and effector markers was: Lymphocytes> Single cell x2> autofluorescent-> Live> CD3e- CD19-> Ly6G- Ly6C+/- (non-neutrophils)> NK1.1+> CD11b+> CD107a+ or Perforin+ or IFNγ+. Frequencies of flow cytometric data are combined from 2 independent experiments, gMFI's are representative images from 2 independent experiments at each dose. Each symbol represents an individual mouse. Mock mice are a mixture of all 3 genotypes. p-values from One-way ANOVA with Tukey's Multiple Comparisons are shown for each panel. *$p<0.05$, **$p<0.01$, ***$p<0.001$, ****$p<0.0001$.

bioRxiv preprint doi: https://doi.org/10.1101/2025.07.15.664967; this version posted July 18, 2025. The copyright holder for this preprint (which was not certified by peer review) is the author/funder, who has granted bioRxiv a license to display the preprint in perpetuity. It is made available under aCC-BY 4.0 International license.

106 **Fig. S6**

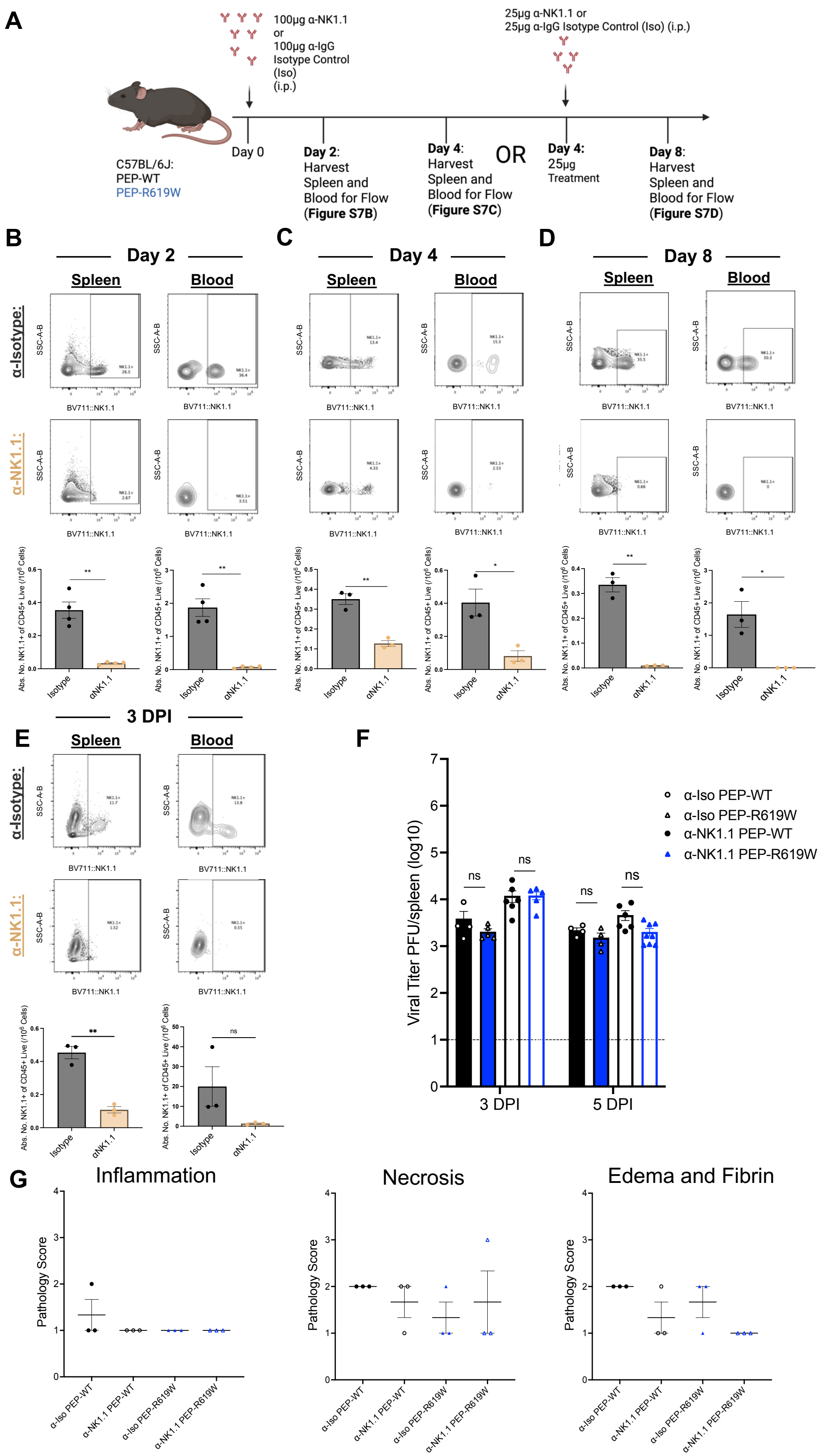

bioRxiv preprint doi: https://doi.org/10.1101/2025.07.15.664967; this version posted July 18, 2025. The copyright holder for this preprint (which was not certified by peer review) is the author/funder, who has granted bioRxiv a license to display the preprint in perpetuity. It is made available under aCC-BY 4.0 International license.

Figure S6: NK depletion protocol and NK-depleted spleen titer and histopathology scores.

Experimental timeline detailing the concentration of α-NK1.1 antibody (Clone PK136) and frequency of doses (A). NK cell depletion representative flow plots and absolute counts of NK1.1+ cells (per 10^6 cells) are shown for the spleen and peripheral blood at day 2 (B) and day 4 (C) post-100µg α-NK1.1 or α-Iso injection (i.p). Alternatively, mice were administered 100µg α-NK1.1 or α-Iso injection (i.p), and on day 4, an additional dose of 25µg α-NK1.1 or α-Iso injection was given. NK cell depletion representative flow plots and absolute counts of NK1.1+ cells (per 10^6 cells) are shown for the spleen and peripheral blood at day 8 (D). NK depletion at 3 dpi following 1e5 PFU MHV A59 infection using the timeline depicted in Figure 6A (E). Following 5e2 PFU MHV A59 infection (i.p), spleens were harvested and tittered at the indicated days post-infection with NK depletion (α-NK1.1) or non-depleting isotype control (α-Iso), with timeline depicted in Figure 6A (F). Liver histopathology scores of individual parameters (inflammation, necrosis and edema/fibrin) are shown (G). PEP-WT mice were used to verify NK cell depletion. The gating strategy to identify splenic and peripheral blood NK cells was: Lymphocytes> Single cell x2> autofluorescent-> Live> CD45+> CD3e- CD19-> NK1.1+. Representative flow plots are shown from CD3e- CD19. Each dot represents an individual mouse. p-values from Welch's unpaired t test are

bioRxiv preprint doi: https://doi.org/10.1101/2025.07.15.664967; this version posted July 18, 2025. The copyright holder for this preprint (which was not certified by peer review) is the author/funder, who has granted bioRxiv a license to display the preprint in perpetuity. It is made available under aCC-BY 4.0 International license.

shown for NK cell counts, *p<0.05, **p<0.01. Viral titers are pooled data from 2 independent experiments at each time point; comparisons are shown from multiple unpaired T-Tests, α-Iso PEP-WT vs α-Iso PEP-R619W, α-NK1.1 PEP-WT vs α-NK1.1 PEP-R619W. For histopathological analysis, each dot represents an individual mouse, with p-values from One-way ANOVA with Tukey's Multiple Comparisons shown, *p<0.05, **p<0.01.

bioRxiv preprint doi: https://doi.org/10.1101/2025.07.15.664967; this version posted July 18, 2025. The copyright holder for this preprint (which was not certified by peer review) is the author/funder, who has granted bioRxiv a license to display the preprint in perpetuity. It is made available under aCC-BY 4.0 International license.

Table S1: Flow Cytometry Antibodies

Antigen	Fluorophore	Dilution (1:x)	Company	Clone	CAT / REF	LOT
Ghost Viability	Violet510	1000	TONBO Biosciences	N/A	13-0870-T100	D0870120221133
CD45	Spark+ BUV395	200	BioLegend	30-F11	103192	B428503
CD45	BUV395	200	BD Biosciences	30-F11	564279	3205129
CD3e	PECy5	250	BioLegend	145-2C11	100309	B359890
CD19	BV711	400	BioLegend	6D5	115555	B287454
CD19	APC	250	BioLegend	6D5	115512	B371440
CD19	AF700	250	BioLegend	6D5	115512	B371440
Ly6C	BV785	400	BioLegend	HK1.4	128041	B386418
Ly6G	PE610	200	Invitrogen	1AB-Ly6g	61-9668-80	2445867
Ly6G	BV570	200	BioLegend	1AB	127629	B418454
CD11c	PECy5.5	200	Invitrogen	N418	35-0114-82	2286398
CD11c	APC-Cy7	200	BioLegend	N418	117324	B391502
CD8a	R710	200	TONBO Biosciences	53-6.7	80-0081-U100	C0081010722803
CD8a	AF700	200	BioLegend	53-6.7	100730	B401541
PDCA-1 (CD317)	Pacific Blue	200	BioLegend	927	127108	B302820
PDCA-1 (CD317)	BV421	200	BioLegend	927	127023	B365891
PDCA-1 (CD317)	BV650	200	BioLegend	927	127019	B365769
CD80	BV421	200	BioLegend	16-10A1	104725	B366741
CD86	APC/Cy7	200	BioLegend	GL-1	105029	B349514
CD86	BV605	200	BioLegend	GL-1	105037	B373866
F4/80	Pacific Orange	100	Invitrogen	BM8	MF48030	2209625
F4/80	PE	200	BioLegend	BM8	123109	B223149
F4/80	FITC	200	BioLegend	BM8	123108	B407719
F4/80	APC/Fire 810	200	BioLegend	BM8	123166	B431884
Tim4	PE	150	BioLegend	RMT4-54	130006	B428998
CD11b	PerCPCy5.5	200	TONBO Biosciences	M1/70	65-0112-U100	C0112121919653
CD11b	PerCPCy5.5	200	BioLegend	M1/70	101228	B423267
CD206	PEcy7	200	BioLegend	C068C2	141719	B239603
CD206	APC	150	Invitrogen	MR6F3	17-2061-80	1956852
MHC-II	FITC	150	BioLegend	M5/114.15.2	107606	B398140
NK1.1	FITC	200	BioLegend	PK136	108705	B376169
NK1.1	BV711	200	BioLegend	PK136	108745	B423146
CD49b	FITC	200	eBioscience	DX5	11-5971-82	E023186
CD49b	PE/Dazzle 594	200	BioLegend	DX5	108924	B396304
CD49b	PE	200	BioLegend	DX5	108908	B391901
CD49a	APC/Fire 750	200	BioLegend	HMα1	142610	B420608
CD107a	PEcy7	200	BioLegend	1D4B	121619	B348898
CD107a	BV650	200	BioLegend	1D4B	121645	B424309
CD27	BV605	200	BioLegend	LG.3A10	124249	B408891
CD27	AF700	200	BioLegend	LG.3A10	124240	B329641
IFNg	BV650	100	BioLegend	XMG1.2	505832	B361629
IFNg	PE	100	BioLegend	XMG1.2	505807	B366833
Perforin	Pacific Blue	100	BioLegend	S160009A	154311	B356368
Granzyme B	PE	100	Invitrogen	GB11	12-8899-41	2682463
Granzyme B	FITC	100	BioLegend	GB11	515403	B397296
TNFa	BV605	100	BioLegend	MP6-XT22	506329	B422381
TNFa	PE-Cy7	100	BioLegend	MP6-XT22	506324	B424713
T-bet	BV605	100	BioLegend	4B10	644817	B387805

List of antibodies used in various combinations throughout all experiments. Antigen, fluorophore, dilution (1:x), company, clone, catalog/reference number, and lot number listed.
